# A Vector Nanoplatform for the Bioimaging of Deep-Seated Tumors

**DOI:** 10.32607/actanaturae.27425

**Published:** 2024

**Authors:** E. I. Shramova, S. M. Deyev, G. M. Proshkina

**Affiliations:** Shemyakin-Ovchinnikov Institute of Bioorganic Chemistry, Moscow, Russian Academy of science, Moscow, 117997 Russian Federation; Sechenov First Moscow State Medical University (Sechenov University), Moscow, 119991 Russian Federation; National Research Centre “Kurchatov Institute”, Moscow, 123098 Russian Federation

**Keywords:** bioluminescence resonance energy transfer, DARPins, protein with a large Stokes shift LSSmKate1, epidermal growth factor receptor type 2 HER2, NanoLuc luciferase, molecular targeted bioimaging

## Abstract

Today, in preclinical studies, optical bioimaging based on luminescence and fluorescence is indispensable in studying the development of neoplastic transformations, the proliferative activity of the tumor, its metastatic potential, as well as the therapeutic effect of antitumor agents. In order to expand the capabilities of optical imaging, sensors based on the bioluminescence resonance energy transfer (BRET) mechanism and, therefore, independent of an external light source are being developed. A targeted nanoplatform based on HER2-specific liposomes whose internal environment contains a genetically encoded BRET sensor was developed in this study to visualize deep-seated tumors characterized by overexpression of human epidermal growth factor receptor type 2 (HER2). The BRET sensor is a hybrid protein consisting of the highly catalytic luciferase NanoLuc (an energy donor) and a LSSmKate1 red fluorescent protein with a large Stokes shift (an energy acceptor). During the bioimaging of disseminated intraperitoneal tumors formed by HER2-positive SKOV3.ip1cells of serous ovarian cystadenocarcinoma, it was shown that the developed system is applicable in detecting deep-seated tumors of a certain molecular profile. The developed system can become an efficient platform for optimizing preclinical studies of novel targeted drugs.

## INTRODUCTION

Despite the tremendous progress achieved in cancer treatment thanks to early diagnosis and innovative therapies, cancer remains among the leading causes of death worldwide. Thus, according to the World Health Organization, the incidence of cancer in 2022 stood at 20 million new cases, almost 50% of which (9.7 million) ended in patient death (https://www.who. int/news/item/01-02-2024-global-cancer-burden-growing-- amidst-mounting-need-for-services). Since metastatic spread is the main cause of death for cancer patients, it is important to develop novel model systems and technologies for preclinical studies that would allow one to assess both the tumor progression process and tumor response to therapy.

Current knowledge of the molecular foundations of oncogenesis, which makes tumor profiling (or typing) feasible, drives the development of targeted therapies selectively addressing particular molecular targets specific to a given cancer type or subtype: cell surface antigens, growth factors, receptors, or signal transduction pathways, which regulate the cell cycle, proliferation, metastatic spread, and angiogenesis.

Along with advances in tumor molecular profiling techniques, preclinical techniques of non-invasive targeted molecular imaging of tumors and metastases are undergoing intensive development in experimental oncology [1, 2, 3]. *In vivo *monitoring of the spread of cell populations exogenously introduced into a model organism is crucial for understanding oncogenesis as well as assessing the therapeutic effect of antitumor agents in preclinical pharmacological research [2, 4].

Whole-body real-time optical bioimaging based on fluorescent and luminescent systems is an indispensable tool in modern preclinical studies [1, 3, 5].

Bioluminescence imaging is based on the detection of visible light emitted as a result of the oxidation of a specific substrate by luciferase [6]. In order to monitor tumor growth or regression, as well as assess the* in vivo *effectiveness of an antitumor drug, the luciferase gene is either constitutively or inducibly expressed in tumor cells that are further used to form the animal model of the cancer [7, 8]. Bioluminescence imaging is widely employed in preclinical studies, but the introduction of this method into clinical practice is being hindered by the fact that the cell line transfected with the luciferase gene needs to be the end product.

Fluorescence imaging allows one to visualize a tumor by detecting light generated by fluorescent proteins, quantum dots, or fluorescent dyes [1]. However, the need for an external light source in order to excite a fluorescent tag imposes significant limitations on the application of this method in detecting deep-seated tumors: as exciting light passes through tissues, its intensity drops abruptly because of diffraction, which reduces the spatial resolution of fluorescence images, as diffusion causes light scattering by tissues, as well as photon absorption by biological chromophores (melanin, hemoglobin, and oxyhemoglobin) [1, 9, 10].

The aforementioned limitations can be overcome using optical bioimaging methods based on the resonance energy transfer mechanism: bioluminescence resonance energy transfer (BRET) or fluorescence resonance energy transfer (FRET), which are increasingly employed in preclinical studies [11]. Although BRET and FRET systems rely on the same mechanism: (Förster resonance energy transfer from donor to acceptor) [12], BRET systems are preferred because the absence of autofluorescence and photobleaching associated with fluorophore excitation ensures increased detection sensitivity at the whole-body level.

The conventional BRET systems consist of luciferase, which acts as a resonance energy donor in the presence of its bioluminescent substrate, and an acceptor represented by a fluorescent protein, dye, or quantum dots. For optical bioimaging in animals to be efficient, a BRET system needs to possess such properties as high energy transfer from a donor to an acceptor and excellent spectral resolution; furthermore, it needs to contain an acceptor emitting in the red spectral region. The red and near-infrared spectral regions are predilected in imaging deep tissues and the whole body, as there is no light absorption by hemoglobin, melanin, and water in this spectral region.

Approximately two dozen high-sensitivity BRET systems have been developed [11]. They employ luciferase from coral *Renilla reniformis *(RLuc), the North American firefly *Photinus pyralis *(Fluc), and genetically engineered NanoLuc luciferase from the deepsea shrimp *Oplophorus gracilirostris *as energy donors, as well as proteins of different colors, including those whose emission maximum lies in the red spectral region [13, 14, 15, 16, 17, 18, 19] as acceptors.

In all the aforementioned studies focusing on the development of BRET sensors based on fluorescent proteins, tumor models comprising genetically engineered cells that stably express the BRET sensor gene were used to monitor tumor cells in an animal body. In this study, we propose a different approach which involves detection of deep-seated tumors in an animal body using a BRET sensor exogenously introduced into the body and exhibiting tropicity for tumors with a given molecular profile.

We chose the tumor-associated antigen HER2 (human epidermal growth factor receptor type 2) as a target. It is known that 15–20% of human breast and ovarian tumors are characterized by an upregulated* HER2 *expression [20, 21]. In modern medical practice, the HER2 tumor marker is a therapeutic target for monoclonal antibodies (Pertuzumab and Trastuzumab) and kinase inhibitors (Lapatinib) in patients with HER2-positive breast tumors [22].

**Fig. 1:**
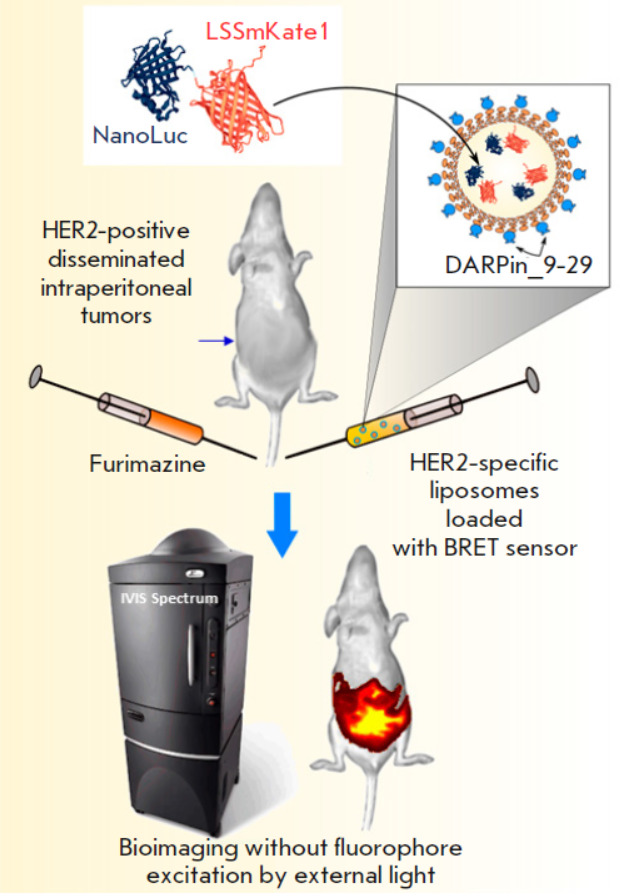
Targeted nanoplatform based on the NanoLuc-LSSmKate1 BRET sensor and HER2-specific liposomes for the non-invasive diagnosis of deep-seated tumors. Conceptual scheme of the experiment: the genetically encoded NanoLuc-LSSmKate1 BRET sensor is incorporated into liposomes whose outer surface is modified with the DARPin_9-29 HER2-specific module. In the presence of a luciferase substrate in the animal body, the red fluorescent protein is activated without an external light source, allowing intravital real-time detection of deep-seated tumors in the animal body

In this study, we designed a platform for detecting HER2-positive tumors based on tumor-specific liposomes loaded with a genetically encoded BRET sensor (*Fig. 1*). The BRET sensor is NanoLuc-LSSmKate1, a hybrid protein based on the highly catalytic NanoLuc luciferase and the large Stokes shift red fluorescent protein LSSmKate1 (λex/λem = 463/624 nm) [23]. In the presence of a substrate, furimazine, NanoLuc luciferase acts as a source of endogenous bioluminescence, thus becoming an energy source for exciting the LSSmKate1 red fluorescent protein. The tropicity of liposomes for the HER2 antigen on the tumor cell surface is determined by the HER2-specific protein DARPin_9-29 [24]. The *in vivo *functionality of the developed system was demonstrated experimentally using the model of deep-seated disseminated tumors.

## EXPERIMENTAL

**Cloning the *NanoLuc-LSSmKate1 *gene and production of the NanoLuc-LSSmKate1, NanoLuc, and DARPin_9-29 proteins**

The nucleotide sequence encoding *LSSmKate1 *was obtained by introducing K69Y/P131T/S148G/M167E/T183S/M196V mutations into the *mKate2 *coding sequence (plasmid pmKate2-N, Evrogen, Russia). The sequences encoding NanoLuc luciferase and the LSSmKate1 red fluorescent protein were then merged in one reading frame and cloned into the pET22b vector. A linker encoding the GGGGS polypeptide inserted between the coding sequences of the *NanoLuc *and* LSSmKate1 *genes. The peptide linker ensured that the two functional domains (NanoLuc luciferase and the LSSmKate1 fluorescent module) in the hybrid protein were not sterically hindered, and that they were able to retain their functional properties while being brought closer together for efficient BRET.

The fidelity of the final construct was verified by sequencing. The coding sequence of the* NanoLuc-LSSmKate1 *gene corresponds to a protein with the following primary structure: MVFTLEDFV GDWRQTAGYNLDQVLEQGGVSSLFQNLGVSVTPI QRIVLSGENGLKIDIHVIIPYEGLSGDQMGQIEKIF KVVYPVDDHHFKVILHYGTLVIDGVTPNMIDYFG RPYEGIAVFDGKKITVTGTLWNGNKIIDERLINPD GSLLFRVTINGVTGWRLCERILAGGGGSMVSELIK ENMHMKLYMEGTVNNHHFKCTSEGEGKPYEGTQ TMRIKVVEGGPLPFAFDILATSFMYGSYTFINHTQ GIPDFFKQSFPEGFTWERVTTYEDGGVLTATQDTS LQDGCLIYNVKIRGVNFTSNGPVMQKKTLGWEA GTEMLYPADGGLEGRSDEALKLVGGGHLICNLKS TYRSKKPAKNLKVPGVYYVDRRLERIKEADKETY VEQHEVAVARYCDLPSKLGHKLNAAALEHHHHHH.

The proteins (NanoLuc-LSSmKate1, NanoLuc, and DARPin_9-29) used in this study were produced by auto-induction [25]. *E. coli *BL21(DE3) colonies transformed with pET22-NanoLuc-LSSmKate1, pET22-NanoLuc, or pET22-DARP were cultured in a ZYM-5052 medium for autoinduction in the presence of ampicillin (100 μg/mL) at 25°C and 200 rpm overnight. The autoinduction medium containing equimolar concentrations of sodium hydrogen phosphate and potassium dihydrogen phosphate prevents acidification of the culture medium by bacterial metabolic products and ensures that neutral pH values are maintained even for high cell-density cultures (*OD*600 ~ 10). Balanced concentrations of glucose, lactose, and glycerol, as well as the high intensity of culture stirring (200 rpm), make it possible to automatically induce gene expression of the target protein (upon glucose depletion in the medium) without controlling the culture density. Biomass was precipitated by 15-min centrifugation at 6,000 *g*, resuspended in 20 mM NaPi, pH 8.0, 150 mM NaCl, and lysozyme (30 μg/mL). Cells were disrupted by ultrasonication; debris was removed by high-speed centrifugation (25,000 *g*). Imidazole was added to the clarified lysate to a final concentration of 30 mM. The lysate was filtered through a membrane (pore diameter, 0.2 μm) and applied onto a 1 mL HisTrap column (Cytiva). Proteins were isolated according to the manufacturer’s protocol. Protein concentrations were determined spectrophotometrically according to the Beer–ambert law using the following extinction coefficients: NanoLuc-LSSmKate1, λ280 = 54570 M-1cm-1; NanoLuc, ε280 = 25400 M-1cm-1; and DARPin, ε280 = 4470 M-1cm-1. Extinction coefficients were determined using the ProtParam tool software (https://web.expasy.org).

**Quantification of BRET efficiency in the NanoLuc-LSSmKate1 system**

The luminescence spectra of NanoLuc-LSSmKate1 and NanoLuc in the presence of 5 μM furimazine were recorded to evaluate BRET efficiency in the NanoLuc-LSSmKate1 system. The measurements were performed 10s after the addition of the luciferase substrate to an IVIS Spectrum CT system (PerkinElmer, USA) in the excitation block mode; the emission spectrum was recorded in the wavelength range of 500–40 nm with an increment of 20 nm. BRET efficiency was calculated as the ratio between the energies emitted by the acceptor (NanoLuc- LSSmKate1) and the donor (NanoLuc) [26, 27].

**Production of HER2-specific liposomes loaded with NanoLuc-LSSmKate1**

NanoLuc-LSSmKate1 was encapsulated into liposomes according to the procedure described in ref. [28]. A phospholipid suspension (0.3 mL, final concentration of 4 g/L) prepared from L-α-phosphatidylcholine pellets (Avanti Polar Lipids, Soy 40%) was mixed with 0.2 mL of NanoLuc-LSSmKate1 (final concentration, 150 μM in 20 mM NaPi, pH 6.0). Encapsulation was based on electrostatic interaction between the positively charged polyhistidine tag on the protein (p*K*a of histidine’s imidazole ~ 6) and the negatively charged inner liposome membrane at neutral pH. The suspension consisting of phospholipids and NanoLuc- LSSmKate1 was subjected to five cycles of rapid freezing (–150°C) and thawing (+30°C), followed by extrusion through a filter with 100-nm pores. The free protein and lipids were separated from the liposomes by gel permeation chromatography on a column packed with the Sepharose CL-2B sorbent.

The outer surface of the liposomes was functionalized with HER-2-specific DARPin_9-29 at the amino groups of phosphotidylethanolamine. For this purpose, the liposomes, loaded with NanoLuc-LSSmKate1, were incubated in the presence of a tenfold molar excess of sulfo-EMCS (N-ε-maleimidocaproyloxysul fosuccinimide ester). Simultaneously, DARPin_9-29 (100 μM in 20 mM NaPi, pH 7.5) was incubated with 2-iminothiolane (6 mM, Traut’s reagent that allows for insertion of the SH group at primary amines of the protein). Both reactions were conducted at room temperature for 40 min; the products were then separated from non-bound modifying agents on a NAP5 column (Cytiva). Conjugation of sulfo-EMCS-proteoliposomes to DARPin-SH was performed during 40 min at room temperature; DARPin-Lip(NanoLuc-LSSmKate1) was separated from non-bound DARPin_9-29 by gel permeation chromatography on a Sepharose CL-2B packed column.

**Cell lines**

A SKOV3.ip1 ovarian serous cystadenocarcinoma cell line derived from the intraperitoneal ascitic fluid of an immunodeficient mouse, which was intraperitoneally injected with SKOV3 human ovarian adenocarcinoma cells [29], as well as a SKOV3.ip1-NanoLuc cell line stably expressing the NanoLuc luciferase gene (collection of cell lines of the Laboratory of Molecular Immunology, Institute of Bioorganic Chemistry RAS), was used in this study. SKOV3.ip1 and SKOV3.ip1-NanoLuc are characterized by overexpression of the HER2 receptor (106 receptors/cell). Cells were cultured under standard conditions (37°C in a humidified atmosphere containing 5% CO_2_) in RPMI 1640 (PanEco, Russia) supplemented with 2 mM L-glutamine (PanEco), 10% fetal bovine serum (Gibco), and an antibiotic (10 U/mL penicillin, 10 μg/mL streptomycin, PanEco).

**Flow cytometry**

The functional activity of the DARPin_9-29 targeted module within the liposomes was studi e d by a s s e s s i n g t h e i nt e r a c t i o n b e t we e n DARP-Lip(NanoLuc-LSSmKate1) and HER2-positive SKOV3.ip1 cells using flow cytometry. Cells (100,000 cells in 200 μL of the complete growth medium) were incubated at 37°C for 10 min in the presence of 300 nM DARP- Lip(NanoLuc-LSSmKate1) (concentration specified for NanoLuc-LSSmKate1). The cells were washed thrice with phosphate-buffered saline and analyzed on a NovoCyte 3000 flow cytometer. LSSmKate1 fluorescence was excited using a 488 nm laser and detected at 615 ± 20 nm (PerCP-H channel).

**Confocal microscopy**

d i n g o f t h e t a r g e t e d m o d u l e w i t h i n DARP-Lip(NanoLuc-LSSmKate1) to the HER2 receptor on the SKOV3.ip1 cell surface was studied by confocal microscopy. For this purpose, 4,000 SKOV3.ip1 cells were inoculated into the wells of a 96-well glass-bottom microplate (Eppendorf) and cultured overnight. The next day, 300 nM of DARP-Lip(NanoLuc-LSSmKate1) was added to the cells (concentration specified for NanoLuc- LSSmKate1). The cells with the conjugate were incubated for 20 and 90 min. Nuclei were stained with 10 nM of the Hoechst 33342 dye for 10 min at 37°C. The cells were washed thrice with phosphate-buffered saline; after addition of the FluoroBright medium (Gibco), the cells were analyzed on an LSM 980 confocal microscope (Carl Zeiss) using a 63× Plan- Apochromat oil immersion lens. The fluorescence of the Hoechst 33342 dye was excited using a 405 nm laser and detected at 410–20 nm; LSSmKate1 was excited using a 488 nm laser, and fluorescence was detected in the wavelength range of 600–55 nm.

**Bioluminescence imaging in the animals**

*In vivo *studies were carried out using Balb/c nude/ nude mice. Experiments involving laboratory animals were performed in compliance with the principles of humane animal treatment as specified in the European Union Directives (86/609/ ECC) and the Declaration of Helsinki, in accordance with the Guidelines for Proper Conduct of Animal Experiments (Protocol of the Committee Controlling Animal Housing and Use of the Institute of Bioorganic Chemistry, RAS, No. 368/2022 dated December 19, 2022). The model of disseminated intraperitoneal metastases was obtained by intraperitoneal inoculation of 2 × 10^6^ SKOV3.ip1-Nano- Luc cells in 100 μL of a serum- and antibiotic-free culture medium. Growth of intraperitoneal tumors was assessed according to the luminescence signal. For this purpose, 7 μg of furimazine (Nano-Glo, Promega) in 100 μL of PBS was injected into the retro-orbital sinus of mice 10 days after inoculation, and bioimaging was performed on an IVIS Spectrum CT system (Perkin Elmier) in the luminescence mode. Fluorescence bioimaging of intraperitoneal tumors was conducted in the epifluorescence mode in the wavelength range of 600–40 nm (with an increment of 20 nm) without any excitation light (the excitation block mode); the agents injected to mice into different retro-orbital sinuses were as follows: 60 min before anesthesia, 2 μM DARP-Lip(NanoLuc- LSSmKate1) (concentration specified for NanoLuc- LSSmKate1); 30 s before anesthesia, and 7 μg of furimazine. Imaging was carried out immediately after the animals had fallen asleep.

## RESULTS AND DISCUSSION

**Fig. 2:**
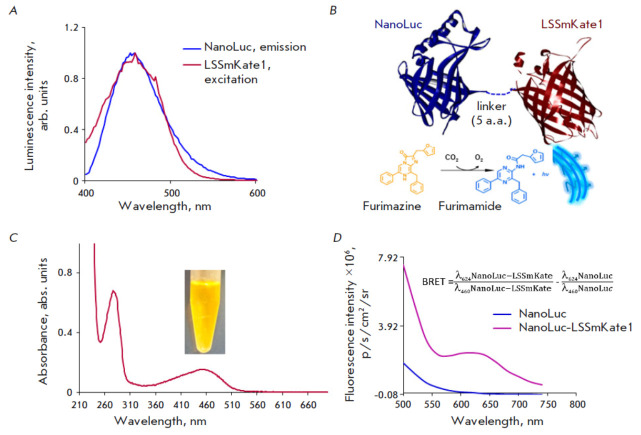
Characteristics of the NanoLuc-LSSmKate1 BRET sensor. (*A*) – Normalized luminescence spectra of NanoLuc luciferase in the presence of 30 μM furimazine (blue curve) and LSSmKate1 fluorescence (dark red curve). (*B*) – Schematic representation of the NanoLuc-LSSmKate1 BRET sensor and the concept at work: NanoLuc luciferase highly specifically oxidizes its substrate furimazine, whose oxidized form, furimamide, emits light in the blue spectral region. Some of this energy is nonradiatively transferred to LSSmKate1 located in the same polypeptide chain as NanoLuc luciferase. LSSmKate1 begins to fluoresce. (*C*) – Absorption spectrum of the purified NanoLuc-LSSmKate1 protein and a protein sample *in vitro*. (*D*) – Fluorescence spectra of NanoLuc-LSSmKate1 (lilac curve) and NanoLuc (blue curve) recorded in the presence of a luciferase substrate on an IVIS Spectrum CT system without excitation by external light (the excitation block mode). A formula for calculating the efficiency of the resonance energy transfer in the NanoLuc-LSSmKate1 system is provided

Among all the luciferases currently used in BRET sensors, NanoLuc is an ideal energy donor, as it stands out for its extraordinary luminance (luminescence intensity) and small size [30]. The LSSmKate1 red protein with a large Stokes shift having an emission maximum at 624 nm was chosen as the energy acceptor [23]. This protein meets two important conditions: (1) the excitation spectrum of LSSmKate1 (excitation maximum, 463 nm) coincides with that of the oxidized form of the luciferase substrate (emission maximum, 460 nm) (*Fig. 2A*); (2) the emission spectrum of LSSmKate1 lies in the transparency window of biotissue (600–1000 nm), where the absorption coefficient of tissue is minimal [31].

BRET efficiency is known to depend on distance: for nonradiative energy transfer to be efficient, the distance between a donor and an acceptor should be ≤ 10 nm [32]. That is why it seemed reasonable to obtain the NanoLuc-LSSmKate1 hybrid protein carrying functional modules (luciferase and fluorescent protein) arranged as close as possible. The scheme of BRET sensor operation is shown in *Fig. 2B*: NanoLuc luciferase oxidizes the furimazine substrate, which emits photons in the visible spectral region when converted to its oxidized form, furimamide. This energy is partially absorbed by the acceptor, the LSSmKate1 fluorescent protein, which then becomes excited and fluoresces.

The NanoLuc-LSSmKate1 construct and the respective protein were prepared according to the procedure described in the Experimental section. The absorption spectrum of the purified NanoLuc- LSSmKate1 protein is characterized by strong absorption in the visible spectral region, as indicated by the presence of a peak at 460 nm and the bright yellow color of the purified protein (*Fig. 2C*).

The efficiency of resonance energy transfer in the NanoLuc–LSSmKate1 system, calculated as the ratio between the emission of the donor–acceptor system (NanoLuc–LSSmKate1) at the emission maximum wavelength of the acceptor (624 nm) and the emission of this system at the emission maximum wavelength of the donor (NanoLuc, 460 nm) minus the same ratio detected for the donor only [8, 33], was equal to 0.3 (*Fig. 2D*).

To selectively deliver the BRET sensor to HER2- positive tumors, we used liposomes whose outer surface was modified with the HER2-specific module DARPin_9-29 (Designed Ankyrin Repeat Proteins), which interacts with subdomain I of the HER2 receptor with high affinity (*K*D = 3.8 nM) [24]. DARPin proteins belong to a new class of targeted non-immunoglobulin- based molecules. These molecules differ from antibodies by their high expression level, monomericity in solutions, small size, resistance to proteases, and high solubility [34, 35]. These features allow DARPins to compete with antibodies as alternative targeted components within multifunctional compounds designed for cancer therapy.

**Fig. 3:**
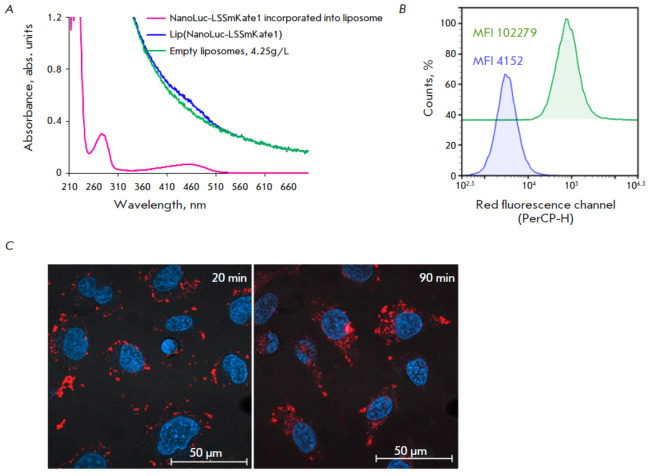
Characteristics of HER2-specific liposomes loaded with the NanoLuc-LSSmKate1 BRET sensor. (*A*) – Absorption spectra of empty (green curve) liposomes and those containing NanoLuc-LSSmKate1 (blue curve). The purple curve corresponds to the NanoLuc-LSSmKate1 protein loaded into liposomes. (*B*) – Flow cytometry data on the receptor-specific interaction of DARP-Lip(NanoLuc-LSSmKate1) with HER2-positive SKOV3ip cells. The blue curve corresponds to the auto-fluorescence of the cells (control), and the green curve corresponds to cells treated with DARP-Lip(NanoLuc-LSSmKate1). Mean fluorescence intensities (MFI) are shown in the pictogram. The signal was detected in the red fluorescence channel (PerCP-H, λem = 615 ± 20 nm) under laser excitation at 488 nm. (*C*) – Merged confocal images in the blue (λex = 405 nm, detection 410–520 nm) and red (λex = 488 nm, detection 600–755 nm) fluorescence channels of SKOV3ip cells after 20-min (left photo) and 90-min (right image) incubation with DARP-Lip(NanoLuc-LSSmKate1). Nuclei are stained with Hoechst33342

The method of loading liposomes with the BRET sensor is based on the electrostatic interaction between the positively charged polyhistidine tag (p*K*a of histidine’s imidazole ~ 6) and the negatively charged inner liposome membrane at neutral pH [28]. The concentration of liposomes loaded with NanoLuc– LSSmKate1 was quantified spectrophotometrically by comparing the absorption spectrum of empty liposomes and that of proteoliposomes. As shown in *Fig. 3A*, the spectrum of proteoliposomes (blue curve) coincides with that of the empty liposomes with a concentration of 4.25 mg/mL (green curve) obtained by passing the phospholipid suspension through a filter with a 100 nm pore diameter 15 times. Previously, we found using the hydrophilic membrane-permeable dye, copper phthalocyanine-3,4’,4’,4’,4’-tetrasulfonic acid tetrasodium salt (CPTS), that the concentration of lipid vesicles in 1 mg/mL suspension corresponds to 1.2 nM [28]: hence, the molar concentration of 4.25 mg/mL of the liposome suspension is 5.1 nM. Subtraction of the spectrum of empty liposomes (green curve in *Fig. 3A*) from that of the liposomes loaded with NanoLuc–LSSmKate1 (blue curve in *Fig. 3A*) yields the spectrum of NanoLuc–LSSmKate1 encapsulated into the liposome (lilac curve in *Fig. 3A*). The concentration of the protein encapsulated into liposomes is ~ 5.42 μM (*OD*280/ε280 = 0.296/54570). Therefore, a single proteoliposome contains ~ 1063 BRET sensor molecules.

Functionalization of proteoliposomes with the DARPin targeted module was conducted using Trout’s reagent (2-iminothiolane) and the hydrophilic amino/sulfhydryl crosslinking agent sulfo- EMCS, according to the procedure described in the Experimental section.

The ability of liposomes loaded with the BRET sensor and functionalized with the DARPin targeted module to interact with the HER2 receptor *in vitro* was studied by flow cytometry and confocal microscopy (*Fig. 3B,C*). The flow cytometry data prove the specific interaction between DARPin-modified liposomes and the HER2 receptor on the SKOV3.ip1 cell surface. As shown in *Fig. 3B*, the mean fluorescence intensity (MFI) of HER2-positive SKOV3.ip1 cells treated with DARP-Lip(NanoLuc-LSSmKate1) is 102,279 (green curve in *Fig. 3B*), which is approximately 25-fold higher than the autofluorescence of these cells (blue curve in *Fig. 3B*).

Confocal microscopy revealed that during a 20-min incubation of SKOV3.ip1 cells in the presence of 300 nM of the DARP-Lip(NanoLuc-LSSmKate1) suspension, targeted proteoliposomes efficiently bind to the cell membrane (the red “crown” along the cell membrane in the left image in *Fig. 3C*). Further incubation for 1.5 h results in internalization of DARP-Lip(NanoLuc-LSSmKate1) as indicated by the red pixels in the cytoplasm (*Fig. 3C*, right image).

Hence, as one can see from the data reported above (*Fig. 3*), the developed system is characterized by a high degree of BRET sensor loading into liposomes and high specificity to the HER2 target.

**Fig. 4:**
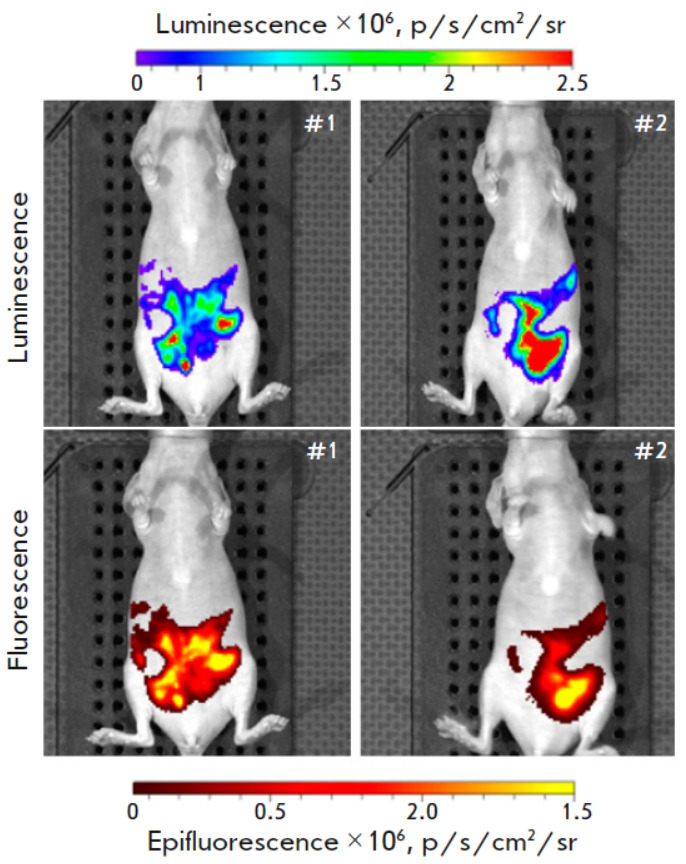
HER2-specific liposomes loaded with the Nano- Luc-LSSmKate1 BRET sensor in the optical bioimaging of disseminated intraperitoneal tumors. The real-time intravital luminescent (top) and fluorescent (bottom) images of animals recorded on an IVIS Spectrum CT system are presented. The images were obtained in two different signal detection modes: top photos, in the bioluminescence mode; bottom photos, in the fluorescence mode without fluorophore excitation

The applicability of BRET sensor-loaded DARPinmodified liposomes in the real-time non-invasive *in vivo *detection of HER2-positive deep-seated tumors was assessed in the mouse model of disseminated intraperitoneal metastases, based on human ovarian carcinoma SKOV3.ip1 cells stably expressing the* NanoLuc *reporter gene. SKOV3.ip1 cells possess a high metastatic potential, mimicking the late stage of ovarian cancer with extensive spread of tumor cells to the peritoneal wall and surface of organs when injected intraperitoneally [29]. Intraperitoneal tumor growth was monitored by detecting the luminescent signal 10 days after the inoculation of tumor cells expressing* NanoLuc *to the animals (*Fig. 4*, top images). The biodistribution of the DARP-Lip(NanoLuc- LSSmKate1) liposomes systemically administered into the animal body was monitored by detecting the fluorescent signal, which was recorded in the mode when there was no excitation by an external light (*Fig. 4*, bottom images). *Figure 4* demonstrates that the intensity and topography of the fluorescent signal detected after administration of furimazine to mice completely coincide with those of the fluorescent signal detected in the mode without fluorophore excitation (excitation block) after administration of DARP-Lip(NanoLuc- LSSmKate1) and furimazine to mice. Therefore, the developed HER2-specific liposomes carrying a BRET sensor can be used in intravital optical bioimaging to detect deep-seated tumors possessing a specific molecular profile.

## CONCLUSIONS

The number of clinically ineffective anticancer drugs is much larger than the number of drugs that have proved to be effective in preclinical studies [2, 36]. This fact indicates that novel models and technologies for the preclinical monitoring of the tumor response to treatment need to be developed [36, 37]. The *in vivo *subcutaneous tumor xenograft models widely used in modern experimental studies enable targeted drug screening and can provide data on drug effectiveness, pharmacokinetics, and pharmacodynamics; however, they cannot be used to assess the metastatic potential of a tumor. Orthotopic models allow one to obtain a relevant disease model, but there arises a problem related to the assessment of how much the tumor burdens the body: what if the tumor dimensions cannot be measured using a caliper? It is clear that the value of any preclinical model for assessing the efficacy of antitumor compounds is ultimately determined by its ability to predict the clinical response in humans as accurately as possible. The need for intravital imaging of the events occurring in the animal body during preclinical studies of antitumor drugs has driven the rapid development of optical bioimaging, while advances in tumor molecular profiling methods have laid the groundwork for developing the targeted molecular imaging of tumors.

In this study, we have developed a system that allows real-time non-invasive detection of HER2- positive disseminated intraperitoneal tumors using targeted liposomes loaded with a NanoLuc- LSSmKate1 BRET sensor. The system is characterized by a high degree of BRET sensor loading into the liposome (*Fig. 3*) and a proteoliposome specificity to the HER2 receptor both *in vitro *and *in vivo* (*Fig. 3* and *Fig. 4*); it allows one to perform whole-body non-invasive imaging of tumor processes (*Fig. 4*).

We believe that the developed targeted system for real-time optical bioimaging based on the NanoLuc- LSSmKate1 BRET sensor can become an efficient platform for optimizing preclinical studies of novel targeted drugs. In addition, the elaborated principle of creating a targeted BRET sensor can become a universal platform for non-invasive bioimaging of deep-seated tumors of any molecular profile by simply changing the vector molecule on the liposome surface.

## Abbreviations

BRET: bioluminescence resonance energy transfer
DARPins: designed ankyrin repeat proteins
HER2: human epidermal growth factor receptor 2
LSS protein: large Stokes shift protein
